# Anaemia prevalence and determinants in under 5 years children: findings of a cross-sectional population-based study in Sudan

**DOI:** 10.1186/s12887-020-02434-w

**Published:** 2020-11-30

**Authors:** Khalid Abdelmutalab Elmardi, Ishag Adam, Elfatih Mohamed Malik, Abdalla Ahmed Ibrahim, Asma Hashim Elhassan, Hmooda Toto Kafy, Lubna Mohammed Nawai, Mujahid Sheikhedin Abdin, Stef Kremers

**Affiliations:** 1 Health Information, Monitoring and Evaluation and Evidence Department, Federal Ministry of Health, Khartoum, Sudan; 2 Department of Obstetrics and Gynecology, Unaizah College of Medicine and Medical Sciences, Qassim University, Unaizah, Saudi Arabia; 3 Faculty of Medicine, University of Khartoum, Khartoum, Sudan; 4 Communicable and Non-Communicable Diseases Control Directorate, Federal Ministry of Health, Khartoum, Sudan; 5 Directorate of Pharmacy, Federal Ministry of Health, Khartoum, Sudan; 6 Directorate General of Primary Health Care, Federal Ministry of Health, Khartoum, Sudan; 7 Department of Health Promotion, NUTRIM School of Nutrition and Translational Research in Metabolism, Faculty of Health, Medicine and Life Sciences, Maastricht, The Netherlands

**Keywords:** Anaemia, Haemoglobin, Pre-school children, Urban, Rural, Camps, Sudan, Regression analysis, Survey

## Abstract

**Background:**

Early childhood is an age at risk of anaemia and its deleterious consequences. In Sudan, there is limited evidence on the prevalence and determinant of anaemia in under-five children. This study was conducted in Sudan to assess the prevalence of anaemia in children and to identify its determinants.

**Methods:**

We conducted a household survey involving children aged 6 months to 5 years in November 2016. A representative population was sampled across rural, urban and camps settlements across 18 states in Sudan. We used a pre-designed questionnaire data collection. Haemoglobin (Hb) level and malaria infection were checked. In this cross-sectional study, we dichotomized the outcome variable and performed logistic regression analyses.

**Results:**

A total of 3094 children under 5 years enrolled in the study, 1566 (50.6%) of them were female and 690 (22.3%) of them were under 2 years old. Anaemia prevalence in the whole cohort (6 months - < 5 years) was 49.4% and the mean haemoglobin concentration was 108.1 (standard deviation (SD): 15.4) g/L. The prevalence in younger (6 months - < 2 years) children (61.9%) was higher than in older (2 - < 5 years) children (45.6%) (*p* < 0.001). Severe anaemia (Hb < 70 g/L) prevalence in the whole population was 1.6%. Age (Odds ratio (OR) 2.25, 95% confidence interval (95%CI) 1.75–2.90, *p* < 0.001), type of place of residence (OR 0.37, 95%CI 0.18–0.74, *p* = 0.005), maternal anaemia (OR 1.74, 95%CI 1.39–2.17, *p* < 0.001), and malaria infection (OR 2.82, 95%CI 1.56–5.11, *p* < 0.001) were the identified predictors of anaemia in the whole cohort. In younger children, only the economic class was an anaemia predictor, with a lower anaemia risk among the rich wealth class (OR 2.70, 95%CI 1.29–5.62, *p* = 0.008). However, in older children, three anaemia predictors were identified. These are maternal anaemia (OR 1.79, 95%CI 1.40–2.28, < 0.001), malaria infection (OR 2.77, 95%CI 1.48–5.21, *p* = 0.002), and type of residency (where camps’ residents were less likely affected with anaemia than rural children (OR 0.38, 95%CI 0.17–0.87, *p* = 0.022)).

**Conclusions:**

About half of the under-5 children in Sudan are anaemic, with worse prevalence in younger children. Efforts targeted at improving socio-economic status, decreasing maternal anaemia and childhood malaria infection may mitigate this alarming trend.

**Supplementary Information:**

The online version contains supplementary material available at 10.1186/s12887-020-02434-w.

## Background

Anaemia is a global public health problem. Being one of the most affected population groups, more than 273 million school-age children are suffering from anaemia over the world [1, 2]. Anaemia is associated with age, gender, ethnicity, lower mother education, and low house-hold wealth index [3–6]. It is also associated with malaria and infections [3, 6]. While low-income countries are the most affected, the social and economic impact of anaemia is badly affecting such countries [6–8].

Anaemia is a preventable condition and its control needs reliable data and continuous monitoring and evaluation process at the country level to establish a baseline, identify the barriers, develop sound interventions, and assess the progress [9]. However, in Sudan national-level anaemia prevalence survey is not frequently conducted. Available data on anaemia in children is based on small-scale research, health services reports, and estimation. The most recently available nationally representative prevalence data on childhood anaemia in Sudan, which was obtained via selected states’ survey in 1995, revealed a prevalence of more than 80% [10]. So, measuring the population prevalence of anaemia and its determinants in children will enable the country to update its figure as well as to have a reliable estimation and a better understanding of the problem. This study was conducted in Sudan to assess the level of anaemia in children 6 months to less than 5 years of age and to identify its determinants.

## Methods

A cross-sectional study was conducted from data collected during a household survey. Identifying the prevalence of anaemia in children was a secondary objective of a malaria indicator survey that took place in Sudan in November 2016.

### Study settings

Sudan, the setting of this study, is a low-middle income East African country with a total population of 39.2 million (projected from 2008 census), of whom, 32.7% classified as urban residents [11, 12]. As a result of the civil conflict, some areas in Darfurs, South Kordofan, and Blue Nile states are facing instability and health services accessibility problems. Consequently, a quite high proportion (8%) of the country population are living in internally displaced persons’ (IDPs) camps and experiencing a hard-living condition. On top of that, the country is hosting several refugee camps in many states. The under-five mortality in Sudan is 68.4 per 1000 live births and the infant mortality rate is 52 per 1000 live births. Despite the investment to improve the nutritional status, one-third of the children under the age of 5 years are considered as underweight [13]. The 2012 malaria indicator survey showed a malaria parasite prevalence of 3.3% with huge variation between states [14]. Sudan was classified by the world bank group as a low-income country with US$ 51.8 billion GDP per capita as estimated for 2016 [15].

### Sampling

With a multi-stage cluster sampling technique, the survey covered all the 18 states of the country. Sample size calculation considered the rural/urban population ratio at state levels as well as the population in camps. At the first stage, a total of 509 clusters were identified for the whole country. Out of the total number of clusters calculated (509), 8% was allocated for camps based on their contribution to the country’s population. Then camps’ clusters were allocated to cover all the 8 IDPs camps and 4 randomly selected refugees camps. The remaining clusters were distributed equally to states. Within each state allocated clusters were distributed based on the rural/urban proportion. In each cluster, 20 households were randomly selected. And in each household, all the household members were selected for the survey. For this study, data related to all surveyed children between the age of 6 and 59 months were used in the study analysis.

### Data collection tools and method

A structured pre-coded pre-tested questionnaire was used for data collection by trained health personnel (see supplementary material 1 and 2). A locally developed electronic survey software was used. The survey software was prepared with logical functions for data validation. The survey questionnaire was then uploaded into electronic-web-based data collection tools (personal data assistants (PDAs)). Survey data was attained from the household head whereas data related to the children were obtained from their mothers/guardian. Under asepsis condition, capillary blood samples were taken in the field by laboratory technicians from children and their mothers to be examined for their haemoglobin level as well as malaria infection. Haemoglobin level was measured in the field using a battery-operated device (HemoCue® 301+ analyser). Malaria infection was assessed using malaria rapid diagnostic test (RDT) - Plasmodium falciparum/vivax specific histidine-rich protein II (SD BIOLINE Malaria Ag Pf/Pv® from STANDARD DIAGNOSTICS INC/ SD). Study participants were informed with study objectives, processes and procedures and were assured of their full rights to participate/quit from the study. Based on their voluntary acceptance of participation in the study, written informed consent was taken from study participants and households’ heads. Ethical clearance for this study was obtained from the national ethical and technical review board at the Sudan Federal Ministry of Health.

### Variables

The outcome (dependant variable) of this study is anaemia in children aged 6 to 59 months (dichotomized as anaemic / not anaemic). The WHO haemoglobin level reference value of less than 110 g/L was used to categorize children and their pregnant mothers as anaemic. In non-pregnant mothers, haemoglobin cut off value of < 120 g/L was used to define anaemia. Severe anaemia in children under 5 years was considered when the haemoglobin level was less than 70 g/L [16, 17]. Independent variables, used in this study in categories, include age, gender, type of place of residence, mother education, mother pregnancy status, maternal anaemia, size of the household, wealth status, child has health insurance, tendency of mother to listen to the radio, source of drinking water, type of sanitation facility, history of fever in the last 2 weeks before the survey day, malaria infection, and level of malaria transmission of the settlement area.

### Analysis

IBM SPSS version 21 was used for data analysis. Data were analysed and presented by disaggregating children into two age groups; younger children (of 6 months to less than 2 years) and older children (of 2 to less than 5 years). Anaemia prevalence was described in percentages and the mean haemoglobin level was given (with its standard deviation (SD)) for the different variables for the two age groups as well as for the overall cohort. Statistical differences at a significant p.value of less than 0.05 were assessed using Chi-square test for anaemia prevalence and T-test (ANOVA) for mean haemoglobin level. The magnitude of anaemia problem was classified based on prevalence into mild public health problem (5–19.9%), moderate public health problem (20–39.9%), and severe public health problem (≥40%) [2]. To determine the malaria parasite prevalence, study investigators assessed parasite infection using RDT in children 2 to less than 10 years of the population [18, 19]. States of the country were classified, based on its level of malaria parasite prevalence, as areas of low- (< 10%), moderate- (10- < 50%), or high-malaria transmission (50% or more) [18, 19]. Based on this classification, all states fell under either low or moderate malaria transmission categories. The wealth status of the households was calculated using the principal component analysis (PCA) based on household ownership of durable goods, living condition, and education level of the household head, which then translated into wealth index. The wealth index was thereafter disaggregated into 5 equal quintiles where the lowest quintile denotes for the poorest and the highest quintile denotes for the richest [20].

Then, three logistic regression models were developed to identify determinants of anaemia; the first for the whole cohort (6 months to less than 5 years), the second for the 6 months to less than 2 years age group, and the third for the 2 to under 5 years age group. Anaemia status (anaemia status =1 if the child was anaemic and 0 otherwise) was used as the study outcome. Backward stepwise (likelihood ratio) logistic regression models were built. The models were developed through the entry of all variables that showed a statistically significant level of < 0.1 *p*. value in any one of the two age groups or the whole cohort [21]. Results were presented in terms of odds ratios (OR) together with its 95% confidence intervals (95%CI) and *p*. values. *P*. value of < 0.05 was considered statistically significant.

## Results

This study included a total of 3094 children between the age of 6 months and less than 5 years with 690 (22.3%) children in the age group of 6 months to less than 2 years. The average household size was 5.5 (SD 2.1) members, and girls represent 50.6% (1566) of the total sample. More details about the characteristics of the study population are given in Table 1.

**Table 1:** Characteristics of the study population

Variables			Younger children (6 months - < 2 years)	Older children (2 - < 5 years)	Total population (6 months - < 5 years)
**Sudan**		**n (%)**	**690 (22.3)**	**2404 (77.7)**	**3094**
**Area classification**	Rural	n (%)	494 (22.6)	1692 (77.4)	2186
	Urban	n (%)	151 (21.7)	545 (78.3)	696
	Camps	n (%)	45 (21.2)	167 (78.8)	212
**Gender**	Male	n (%)	325 (21.3)	1203 (78.7)	1528
	Female	n (%)	365 (23.3)	1201 (76.7)	1566
**Mother educational level**	No formal education	n (%)	177 (24.4)	547 (75.6)	724
	Primary education	n (%)	201 (23.0)	673 (77.0)	874
	Secondary education	n (%)	57 (21.7)	206 (78.3)	263
	Above secondary education	n (%)	28 (24.8)	85 (75.2)	113
**Mother frequently listen to the radio**	No	n (%)	330 (23.3)	1086 (76.7)	1416
	Yes	n (%)	130(22.8)	440 (77.2)	570
**Drinking water source**	Piped source to house OR bottled water	n (%)	82 (21.6)	298 (78.4)	380
	Piped source to public area	n (%)	231 (23.1)	771(76.9)	1002
	Open source	n (%)	349 (22.1)	1230 (77.9)	1579
**Sanitation facility type**	Safe sanitation facility	n (%)	93 (22.2)	326 (77.8)	419
	Unsafe sanitation facility	n (%)	347 (21.1)	1299 (78.9)	1646
	Unsafe sanitation - open defecation	n (%)	238 (24.4)	739 (75.6)	977
**Size of the household**	Less than 7 persons	n (%)	510 (22.5)	1754 (77.5)	2264
	7 persons or more	n (%)	180 (21.7)	650 (78.3)	830
**Wealth quintiles**	Lowest	n (%)	158 (23.9)	502 (76.1)	660
	Second	n (%)	148 (22.3)	515 (77.7)	663
	Middle	n (%)	142 (22.4)	491 (77.6)	633
	Fourth	n (%)	131 (20.7)	502 (79.3)	633
	Highest	n (%)	111 (22.0)	394 (78.0)	505
**Child has health insurance**	No health insurance	n (%)	565 (23.9)	1796 (76.1)	2361
	Has health insurance	n (%)	125 (17.1)	608 (82.9)	733
**Pregnancy status of the mother**	Mother not pregnant	n (%)	419 (25.8)	1208 (74.2)	1627
	Mother pregnant	n (%)	34 (11.1)	271(88.9)	305
**Anaemia status of the mother**	Mother not anaemic	n (%)	276 (25.6)	801 (74.4)	1077
	Mother anaemic	n (%)	103 (19.5)	425 (80.5)	528
**Child had fever in the last 2 weeks**	No fever	n (%)	486 (20.3)	1913 (79.7)	2399
	With fever	n (%)	195 (29.1)	474 (70.9)	669
**Child malaria test**	Negative	n (%)	636 (21.9)	2270 (78.1)	2906
	Positive	n (%)	18 (11.9)	133 (88.1)	151
**Level of malaria transmission**	Moderate transmission (PR2–10 = 10- < 50%)	n (%)	117 (21.0)	441 (79.0)	558
	Low transmission (PR2–10 = < 10%)	n (%)	573 (22.6)	1963 (77.4)	2536

n: frequency

### Anaemia prevalence and risk factors in 6 months to under 5 years old children

**Fig. 1:**
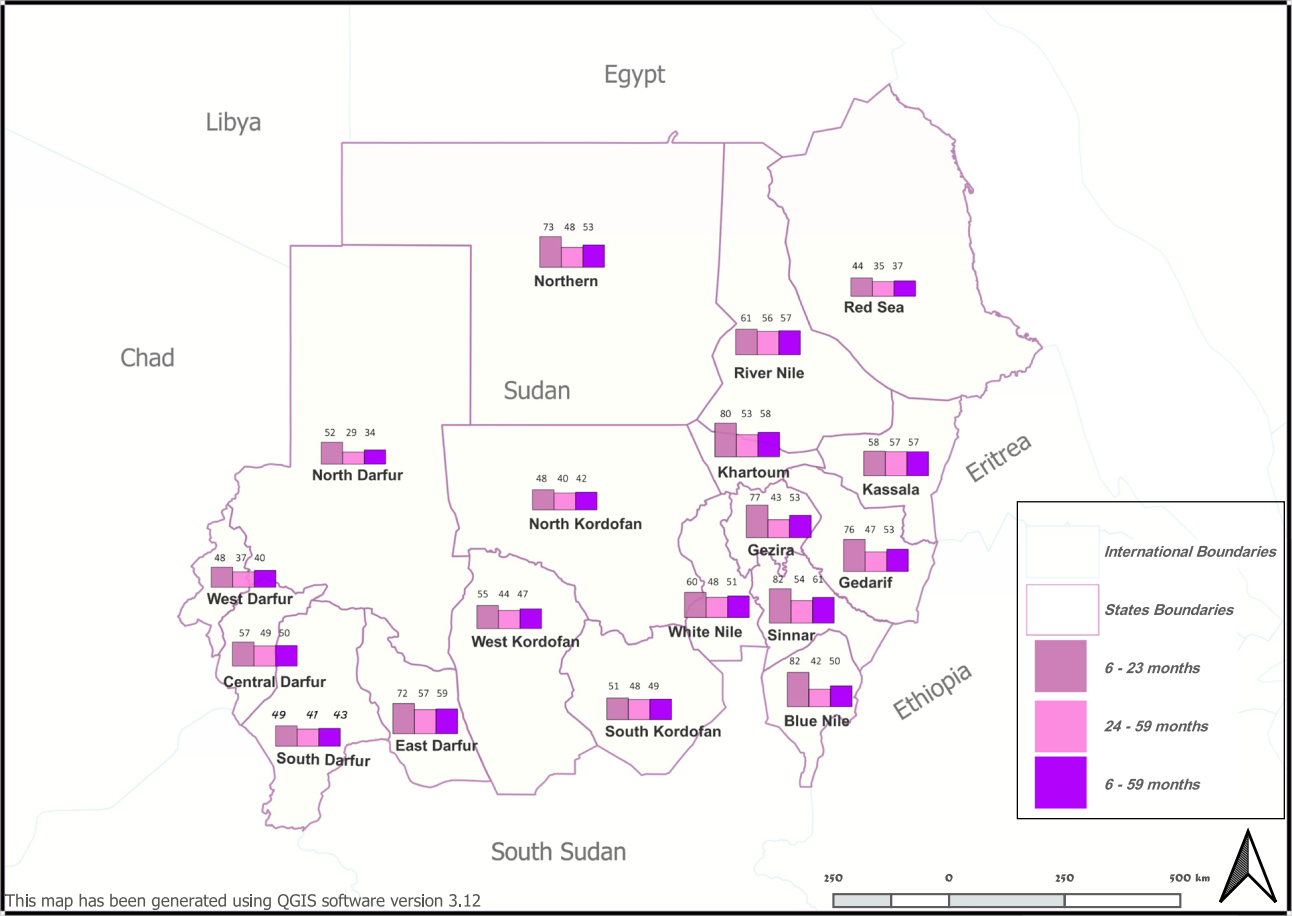
Anaemia prevalence in preschool children by age and states in Sudan, 2016

Tables 2 and 3 show the prevalence of anaemia and the mean haemoglobin level disaggregated by age groups and variables of this study. Overall, both anaemia prevalence (49.4%) and severe anaemia prevalence (1.6%) in Sudanese preschool children were relatively high. The average haemoglobin level (108.1 g/L (SD 15.4)) was below the cut off level for anaemia. The variation in anaemia prevalence between states (range 33.6 to 61.2% (*p* < 0.001)) (Fig. 1) was noticed with similar feature seen for severe anaemia (range: 0.0 and 7.6% (*p* < 0.001)). There were no statistically significant variations in anaemia prevalence (*p* = 0.076) as well as in severe anaemia prevalence (*p* = 0.130) between rural, urban, and camps residents (Table 2). The prevalence of anaemia was higher in the age group of 6 months to less than 2 years compared to the older age group (*p* < 0.001) (Table 3). The trend of higher anaemia prevalence in the younger age group (6 months to < 2 years) was prominent across the majority of the variables in this study. Results also showed that severe anaemia was highly prevalent among children with malaria infection (7.9%) than those without malaria infection (1.3%) (*p* < 0.001), among children living in moderate malaria transmission areas (3.0%) than those in low transmission areas (1.3%) (*p* = 0.008), and in children with a history of fever (3.6%) than those without (1.0%) (*p* < 0.001).

**Table 2:** Anaemia prevalence and haemoglobin level of 6 months to under 5 years old children by age groups, states, and type of place of residence, Sudan, 2016

Variables		Anaemia prevalence in 6 months to under 2 years	Anaemia prevalence in 2 to under 5 years	***p***. value	Anaemia prevalence (overall)	Severe anaemia prevalence (overall)	N
							Mean haemoglobin (g/L)
**Sudan**	Frequency	427/690	1096/2404	–	1523/3094	50/3094	3094
	%	61.9%	45.6%	*< 0.001*	49.2%	1.6%	108.1 (15.4)
***States***							
Northern	Frequency	16/22	39/81	*–*	55/103	1/103	103
	%	72.7%	48.1%	*0.040*	53.4%	1.0%	107.4 (12.8)
River Nile	Frequency	17/28	60/107	*–*	77/135	2/135	135
	%	60.7%	56.1%	*0.659*	57.0%	1.5%	106.0 (15.7)
Red Sea	Frequency	4/9	19/54	*–*	23/63	0/63	63
	%	44.4%	35.2%	*0.593*	36.5%	0.0%	108.2 (13.5)
Kassala	Frequency	30/52	109/190	*–*	139/242	4/242	242
	%	57.7%	57.4%	*0.967*	57.4%	1.7%	105.1 (15.5)
Gedarif	Frequency	29/38	68/146	*–*	97/184	2/184	184
	%	76.3%	46.6%	*0.001*	52.7%	1.1%	108.7 (15.2)
Khartoum	Frequency	32/40	70/131	*–*	102/171	3/171	171
	%	80.0%	53.4%	*0.003*	59.6%	1.8%	104.6 (15.1)
Gezira	Frequency	37/48	52/122	*–*	89/170	0/170	170
	%	77.1%	42.6%	*< 0.001*	52.4%	0.0%	108.2 (12.3)
White Nile	Frequency	22/37	55/114	*–*	77/151	3/151	151
	%	59.5%	48.2%	*0.236*	51.0%	2.0%	107.0 (14.4)
Sinnar	Frequency	31/38	62/114	*–*	93/152	4/152	152
	%	81.6%	54.4%	*0.003*	61.2%	2.6%	103.8 (14.2)
Blue Nile	Frequency	36/44	68/163	*–*	104/207	3/207	207
	%	81.8%	41.7%	*< 0.001*	50.2%	1.4%	107.9 (15.4)
North Kordofan	Frequency	23/48	60/150	*–*	83/198	1/198	198
	%	47.9%	40.0%	*0.333*	41.9%	0.5%	110.2 (14.1)
South Kordofan	Frequency	22/43	72/151	*–*	94/194	2/194	194
	%	51.2%	47.7%	*0.687*	48.5%	1.0%	108.7 (14.1)
West Kordofan	Frequency	16/29	43/98	*–*	59/127	1/127	127
	%	55.2%	43.9%	*0.284*	46.5%	0.8%	109.8 (15.7)
North Darfur	Frequency	26/50	59/203	*–*	85/253	1/253	253
	%	52.0%	29.1%	*0.002*	33.6%	0.4%	114.4 (14.7)
West Darfur	Frequency	28/59	51/138	*–*	79/197	2/197	197
	%	47.5%	37.0%	*0.168*	40.1%	1.0%	111.2 (13.6)
South Darfur	Frequency	28/57	85/206	*–*	113/263	6/263	263
	%	49.1%	41.3%	*0.298*	43.0%	2.3%	109.5 (17.4)
Central Darfur	Frequency	17/30	62/127	*–*	79/157	12/157	157
	%	56.7%	48.8%	*0.439*	50.3%	7.6%	105.4 (20.2)
East Darfur	Frequency	13/18	62/109	*–*	75/127	3/127	127
	%	72.2%	56.9%	*0.220*	59.1%	2.4%	105.6 (16.2)
p. value*		*< 0.001*	*< 0.001*	*–*	*< 0.001*	< 0.001	*< 0.001*
***Area classification***							
Rural	Frequency	308/494	795/1692	*–*	1103/2186	33/2186	2186
	%	62.3%	47.0%	*< 0.001*	50.5%	1.5%	107.9 (15.2)
Urban	Frequency	96/151	231/545	*–*	327/696	10/696	696
	%	63.6%	42.4%	*< 0.001*	47.0%	1.4%	108.6 (15.2)
Camp Refugees	Frequency	23/45	70/167	*–*	93/212	7/212	212
	%	51.1%	41.9%	*0.270*	43.9%	3.3%	109.0 (18.0)
p. value*		*0.295*	*0.106*	*–*	*0.076*	0.130	*0.389*

N: total number of cases included in the analysis

* *p*. value for categorical data (anaemia) is calculated based on Chi-square test and for continuous data (haemoglobin level) is based on ANOVA test

**Table 3:** Anaemia prevalence and haemoglobin level of 6 months to under 5 years old children by age groups risk factors, Sudan, 2016

Variables			Anaemia prevalence in 6 months to under 2 years	Anaemia prevalence in 2 to under 5 years	***p***. value	Anaemia prevalence (overall)	Severe anaemia prevalence (overall)	N
								Mean haemoglobin (g/L)
**Personal factors**	***Age group***							
	6 months - < 2 years	Frequency	NA	NA	NA	427/690	10/690	690
		%	NA	NA	NA	61.9%	1.4%	104.8 (14.5)
	2 - < 5 years	Frequency	NA	NA	NA	1096/2404	40/2404	2404
		%	NA	NA	NA	45.6%	1.7%	109.1 (15.5)
	p. value*		NA	NA	NA	*< 0.001*	0.694	*< 0.001*
	***Gender***							
	Male	Frequency	215/325	580/1203	*–*	795/1528	32/1528	1528
		%	66.2%	48.2%	*< 0.001*	52.0%	2.1%	107.3 (16.1)
	Female	Frequency	212/365	516/1201	*–*	728/1566	18/1566	1566
		%	58.1%	43.0%	*< 0.001*	46.5%	1.1%	109.0 (14.7)
	p. value*		*0.290*	*0.010*	*–*	*0.002*	0.045	*0.002*
**Education factors**	***Mother educational level***							
	No formal education	Frequency	112/177	270/547	*–*	382/724	10/724	724
		%	63.3%	49.4%	*0.001*	52.8%	1.4%	107.2 (14.5)
	primary education	Frequency	127/201	311/673	*–*	438/874	7/874	874
		%	63.2%	46.2%	*< 0.001*	50.1%	0.8%	108.1 (14.4)
	secondary education	Frequency	35/57	91/206	*–*	126/263	3/263	263
		%	61.4%	44.2%	*0.021*	47.9%	1.1%	108.5 (14.1)
	above secondary education	Frequency	17/28	34/85	*–*	51/113	1/113	113
		%	60.7%	40.0%	*0.056*	45.1%	0.9%	110.2 (14.0)
	p. value*		*0.988*	*0.298*	*–*	*0.315*	0.725	*0.141*
	***Mother frequently listen to the radio***							
	No	Frequency	205/330	510/1086	*–*	715/1416	17/1416	1416
		%	62.1%	47.0%	*0.001*	50.5%	1.2%	107.2 (14.5)
	Yes	Frequency	83/130	205/440	*–*	288/570	5/570	570
		%	63.8%	46.4%	*< 0.001*	50.5%	0.9%	108.1 (14.4)
	p. value*		*0.731*	*0.895*	*–*	*0.990*	0.641	*0.867*
**Maternal factors**	***Pregnancy status of the mother***							
	Mother not pregnant	Frequency	260/219	556/1208	*–*	816/1627	19/1627	1627
		%	62.1%	46.0%	*< 0.001*	50.2%	1.2%	108.0 (14.6)
	Mother pregnant	Frequency	22/34	131/271	*–*	153/305	2/305	305
		%	64.7%	48.3%	*0.072*	50.2%	0.7%	108.4 (13.5)
	p. value*		*0.759*	*0.490*	*–*	*0.997*	0.560	*0.649*
	***Anaemia status of the mother***							
	Mother not anaemic	Frequency	165/276	328/801	*–*	493/1077	12/1077	1077
		%	59.8%	40.9%	*< 0.001*	45.8%	1.1%	109.0 (14.3)
	Mother anaemic	Frequency	73/103	234/425	*–*	307/528	6/528	528
		%	70.9%	55.1%	*0.004*	58.1%	1.1%	105.8 (14.2)
	p. value*		*0.047*	*< 0.001*	*–*	*< 0.001*	1.000	*< 0.001*
**Economic factors**	***Size of the household***							
	Less than 7 persons	Frequency	315/510	812/1754	*–*	1127/2264	35/2264	2264
		%	61.8%	46.3%	*< 0.001*	49.8%	1.5%	107.9 (15.4)
	7 persons or more	Frequency	112/180	284/650	*–*	396/830	15/830	830
		%	62.2%	43.7%	*< 0.001*	47.7%	1.8%	109.0 (15.4)
	p. value*		*0.913*	*0.255*	*–*	*0.308*	0.610	*0.079*
	***Wealth quintiles***							
	Lowest	Frequency	89/158	241/502	*–*	330/660	14/660	660
		%	56.3%	48.0%	*0.068*	50.0%	2.1%	107.3 (16.4)
	Second	Frequency	79/148	244/515	*–*	323/663	13/663	663
		%	53.4%	47.4%	*0.198*	48.7%	2.0%	108.0 (15.3)
	Middle	Frequency	95/142	208/491	*–*	303/633	11/633	633
		%	66.9%	42.4%	*< 0.001*	47.9%	1.7%	108.8 (16.0)
	Fourth	Frequency	95/131	236/502	*–*	331/633	5/633	633
		%	72.5%	47.0%	*< 0.001*	52.3%	0.8%	107.9 (14.2)
	Highest	Frequency	69/111	167/394	*–*	236/505	7/505	505
		%	62.2%	42.4%	*< 0.001*	46.7%	1.4%	108.8 (15.0)
	p. value*		*0.006*	*0.203*	*–*	*0.363*	0.343	*0.356*
	***Child has health insurance***							
	No health insurance	Frequency	344/565	839/1796	*–*	1183/2361	39/2361	2361
		%	60.9%	46.7%	*< 0.001*	50.1%	1.7%	107.7 (15.4)
	Has health insurance	Frequency	83/125	257/608	*–*	340/733	11/733	733
		%	66.4%	42.3%	*< 0.001*	46.4%	1.5%	109.5 (15.3)
	p. value*		*0.251*	*0.057*	*–*	*0.078*	0.868	*0.008*
**Hygienic factors**	***Drinking water source***							
	piped source to house OR bottled water	Frequency	53/82	127/298	*–*	180/380	5/380	380
		%	64.6%	42.6%	*< 0.001*	47.4%	1.3%	109.2 (16.0)
	piped source to public area	Frequency	141/231	369/771	*–*	510/1002	18/1002	1002
		%	61.0%	47.9%	*< 0.001*	50.9%	1.8%	107.6 (15.2)
	open source	Frequency	215/349	559/1230	*–*	774/1579	25/1579	1579
		%	61.6%	45.4%	*< 0.001*	49.0%	1.6%	108.1 (15.3)
	p. value*		*0.843*	*0.274*	*–*	*0.446*	0.807	*0.199*
	***Sanitation facility type***							
	safe sanitation facility	Frequency	60/93	128/326	*–*	188/419	5/419	419
		%	64.5%	39.3%	*< 0.001*	44.9%	1.2%	109.0 (15.6)
	unsafe sanitation facility	Frequency	222/347	582/1299	*–*	804/1646	28/1646	1646
		%	64.0%	44.8%	*< 0.001*	48.8%	1.7%	108.4 (15.3)
	unsafe sanitation - open defecation	Frequency	138/238	364/739	*–*	502/977	17/977	977
		%	58.0%	49.3%	*0.019*	51.4%	1.7%	107.4 (15.8)
	p. value*	Frequency	*0.293*	*0.008*	*–*	*0.079*	0.735	*0.140*
**Malaria and infection factors**	***Child had fever in the last 2 weeks***							
	No fever	Frequency	302/486	856/1913	*–*	1158/2399	25/2399	2399
		%	62.1%	44.7%	*< 0.001*	48.3%	1.0%	108.6 (14.7)
	With fever	Frequency	119/195	231/474	*–*	350/669	24/669	669
		%	61.0%	48.7%	*0.004*	52.3%	3.6%	106.7 (17.7)
	p. value*		*0.787*	*0.119*	*–*	*0.064*	< 0.001	*0.004*
	***Child malaria test***							
	Negative	Frequency	399/636	1007/2270	*–*	1406/2906	38/2906	2906
		%	62.7%	44.4%	*< 0.001*	48.4%	1.3%	108.5 (15.2)
	Positive	Frequency	12/18	89/133	*–*	101/151	12/151	151
		%	66.7%	66.9%	*0.983*	66.9%	7.9%	101.0 (18.9)
	p. value*		*0.734*	*< 0.001*	*–*	*< 0.001*	< 0.001	*< 0.001*
	***Level of malaria transmission***							
	Moderate transmission (PR2–10 = 10- < 50%)	Frequency	75/117	202/441	*–*	277/558	17/558	558
		%	64.1%	45.8%	*< 0.001*	49.6%	3.0%	107.5 (16.5)
	Low transmission (PR2–10 = < 10%)	Frequency	352/573	894/1963	*–*	1246/2536	33/2536	2536
		%	61.4%	45.5%	*< 0.001*	49.1%	1.3%	108.3 (15.2)
	p. value*		*0.588*	*0.920*	*–*	*0.828*	0.008	*0.244*

N: total number of cases. *NA* Not applicable

* *p*. value for categorical data (anaemia) is calculated based on Chi-square test and for continuous data (haemoglobin level) is based on ANOVA test

The logistic regression model (Table 4) demonstrated the relationship between anaemia and age with the young children being more prone to anaemia (OR 2.25, 95%CI 1.75–2.90, p < 0.001). The model identified the type of place of residence as a predicting factor of anaemia (*p* = 0.010), a relationship that was not established through the initial binary analysis. The odds ratio was in favour of low anaemia in the camps’ children compared to the rural (OR 0.37, 95%CI 0.18–0.74, *p* = 0.005).

**Table 4:** Regression analysis for anaemia associated factors in 6 months to under 5 years old children by age groups, Sudan, 2016

Variables	Category	Overall (6 months - < 5 years)			6 months - < 2 years age group			2 - < 5 years age group		
		n	aOR (95% CI)	***p***. value*	n	aOR (95% CI)	***p***. value*	n	aOR (95% CI)	***p***. value*
**Age of the child**	2 - < 5 years	1192	1	**< 0.001**	NA	NA	NA	NA	NA	NA
	6 months - < 2 years	347	2.25 (1.75–2.90)	< 0.001	NA	NA	NA	NA	NA	NA
**Area classification**	Rural	1128	1	**0.010**	253	–	NS	879	1	**0.032**
	Urban	370	0.87 (0.66–1.14)	0.314	84	–	–	288	0.85 (0.65–1.11)	0.232
	Camps	41	0.37 (0.18–0.74)	0.005	10	–	–	31	0.38 (0.17–0.87)	0.022
**Anaemia status of the mother**	Mother not anaemic	1025	1	**< 0.001**	252	–	NS	779	1	**< 0.001**
	Mother anaemic	514	1.74 (1.39–2.17)	< 0.001	95	–	–	419	1.79 (1.40–2.28)	< 0.001
**Wealth quintiles**	Lowest	234	–	NS	60	1	**0.003**	174	–	NS
	Second	294	–	–	70	0.73 (0.37–1.50)	0.405	225	–	–
	Middle	270	–	–	60	1.44 (0.68–3.02)	0.344	210	–	–
	Fourth	399	–	–	85	2.70 (1.29–5.62)	0.008	318	–	–
	Highest	342	–	–	72	1.01 (0.51–2.07)	0.937	271	–	–
**Child malaria test**	Negative	1483	1	**< 0.001**	339	–	NS	1150	1	**0.001**
	Positive	56	2.82 (1.56–5.11)	0.001	8	–	–	48	2.77 (1.48–5.21)	0.002

n: number of cases included in the analysis. *aOR* Adjusted Odds Ratio, *NA* Not applicable, *NS p*. value is statistically not significant

*p. values in bold reflect the overall exposure and are derived from the likelihood ratio test. *p*. value for variables with multiple exposure level are reported from the Wald test

Variable included in the models are child age, gender, area classification, sanitation facility type, child fever history in the last 2 weeks, child has health insurance, wealth quintiles, anaemia status of the mother, and child malaria infection. Only variables with statistically significant *p*. values in the final model are presented in this table

The Good-ness-of-fit test (*Hozmer and Lemeshow*) for the adjusted Odds Ratios for the 6 months to < 2 years age group *X*^*2*^ = 1.847 (7 df, *p*. value 0.968), for the 2 to < 5 years group *X*^*2*^ = 2.027 (5 df, *p*. value 0.845), and for the overall (6 months to less than 5 years) *X*^*2*^ = 13.947 (8 df, *p*. value 0.083)

### Anaemia prevalence and risk factors in young children

The prevalence of anaemia in this age group was high (61.9%), with no statistically significant difference in prevalence between boys and girls (*p* = 0.290) (Table 3). Anaemia status was not found to vary statistically with the mother level of education nor with the mother tendency to listen to the radio. Anaemia in children in this age group was not affected by the pregnancy status of their mothers as well. On the other hand, anaemia in early childhood was higher (*p* = 0.047) among children of anaemic mothers (70.9%) compared to those of non-anaemic mothers (59.8%). Neither the household size nor the health insurance status of the children tended to affect the anaemia prevalence among this age group. The anaemia prevalence across the different economic classes varies with higher prevalence in the richest (62.2%) compared to the poorest (56.3%) classes in the younger children. Anaemia was neither found to statistically vary by different sources of drinking water (*p* = 0.843) nor by various types of sanitation facility used (*p* = 0.293). As well, no statistical difference was found between anaemia and children’s history of fever in the last 2 weeks (*p* = 0.787), malaria infection (*p* = 0.734), or level of malaria transmission (*p* = 0.588).

The economic classification was found as a predicting factor of anaemia (*p* = 0.003; Table 4) with a higher risk of anaemia in the second richest class compared to the poorest class in the younger age group (OR 2.70, 95%CI 1.29–5.62, *p* = 0.008). The model showed that all other variables in this study are not predictors of anaemia in children less than 2 years.

### Anaemia prevalence and risk factors in older children

In children of the age group of 2 to less than 5 years old, anaemia prevalence was relatively high (45.6%), with variation in prevalence between boys (48.2%) and girls (43.0%) (*p* = 0.010) (Table 3). There was no statistically significant difference in anaemia prevalence concerning mother education, mother listening to the radio, or maternal pregnancy status. But the prevalence of anaemia was higher (*p* < 0.001) in children whose mothers were anaemic (55.1%) compared to the children whose mothers were not (40.9%). Variation in economic variables included in this study (size of the household (*p* = 0.255), having health insurance for the child (*p* = 0.057), and economic class (*p* = 0.203)) was not found to affect the anaemia prevalence in this age category. Anaemia prevalence in older children did not differ significantly by variations in access to safe sources of drinking water (*p* = 0.274), however, it was higher (*p* = 0.008) in children whose families had access to unsafe sanitation (49.3%) and those who used open defecation areas (44.8%) compared to those who had access to safe sanitation facilities (39.3%). No meaningful difference was found in anaemia prevalence among children with fever and those without fever in the last 2 weeks before the survey (*p* = 0.119), and among older children who live in low malaria transmission areas compared to those living in moderate malaria transmission areas (*p* = 0.920). A higher level of anaemia prevalence was noticed (*p* < 0.001) among older children with malaria infection (66.9%) compared to those who had no malaria infection (44.4%).

The type of place of residence was identified as anaemia predictor in older children (*p* = 0. 032) in the model (Table 4), where the OR was indicative of lower anaemia burden in camps residents compared to rural population (OR 0.38, 95%CI 0.17–0.87, *p* = 0.022). The model also showed that anaemia in older children was predicted by maternal anaemia, where older children of anaemic mothers were more likely to be anaemic (OR 1.79, 95%CI 1.40–2.28, < 0.001). Malaria infection was found as a predictor of anaemia (*p* = 0.001) with older children with malaria infection being at a higher risk of anaemia than those without malaria infection (OR 2.77, 95%CI 1.48–5.21, *p* = 0.002).

## Discussion

We showed that childhood anaemia is highly prevalent in Sudan and about 8% higher than the global prevalence [2]. The problem is severely affecting children resident in rural, urban, and camps areas. There is a noticeable variation in anaemia prevalence between the younger and the older age groups. The study estimated the level of severe anaemia as well. Findings of this study identified the role of age, type of place of residence, maternal anaemia, and malaria infection as anaemia determinants in children between the age of 0.5 to < 5 years. However, different determinants of anaemia were identified for the different age categories. In young children (0.5 to < 2 years), only the economic status was found as a determinant of anaemia. On the other hand, the identified determinants in older children (2 - < 5 years) are the type of place of residence, maternal anaemia, and malaria infection.

The prevalence of anaemia in Sudan is higher than the global and regional estimates for under 5 years old children [2]. The 59% WHO estimation of the anaemia prevalence among children in Sudan is slightly higher than the findings of this study. However, this WHO estimation was for the former Sudan which included the population of the current South Sudan country [2]. Data from Sudan on anaemia prevalence in children is limited. A previous study in a rural area conducted in 2011 in the Northern state showed 80% prevalence of anaemia among pre-school children [22]. Data from the 1995 household survey that covered 6 states of the country, identified anaemia prevalence to range from 82 to 92% in preschool children [10]. The declining trend in anaemia in Sudan based on these comparison needs to be looked at cautiously, since studies’ methodology, coverage and targeted population are different. Preschool children in Ethiopia are also suffering from a similarly high level of anaemia (56%) [23].

In Sudan and as this study showed, the prevalence of severe anaemia in the whole cohort of 1.6% is at a level similar to the global estimate of 1.5% [1]. Findings from a hospital-based study in Sudan showed that severe anaemia among children under 18 years old was 7.8% [24]. This situation urges for action. The higher prevalence of severe anaemia, identified in this study, in children with a history of fever, in children living in a malaria area, and in children with malaria infection could indicate the effect of malaria control, and probably other infections control, on severe anaemia prevention. Nevertheless, severe anaemia is a complex situation that involved many interplaying factors, a situation that needs synergistically working multi-approach actions for its control [25]. Such approaches include disease control, nutritional, and socio-economic interventions.

As Prieto-Patron showed from analysis of 52 Demographic Health Surveys in low and low-middle income countries, anaemia prevalence in children under 2 years old was shown to be 70% [5] which is not too far from the prevalence of 61.9% in this age group in this study. The present study showed a higher risk of anaemia in young children (6–23 months) than in the older children (2- < 5 years) which is supported by others’ findings [3, 7, 26, 27]. From these pieces of evidence, it is clear that the under 5 years old age group is not homogenous in regards to anaemia. So, anaemia prevalence, risk factors and interventions need to be addressed by disaggregating the age of children into two or more age groups. The variation in anaemia causes according to age is also reinforcing this recommendation [4]. However, results in this study may be influenced by the larger sample size of the 2 to < 5 years age group which dominated the findings of the overall cohort.

While the model analysis in this study showed no determination role of gender on anaemia, the role of gender as an anaemia determinant was well documented in many other studies in under 2 years old children which showed a higher risk among boys [3, 5, 28, 29].

The prevalence of anaemia of 43.9% in under 5 years old camps residents in this study looks similar to IDPs (41%) [30] and refugees (47%) [31] camps elsewhere in the same age group. Type of place of residence was found in this study as a predictor of anaemia in the children in the age group of 2 - < 5 years of age, with camps residents being at a lower risk of anaemia. Evidence from other studies showed no rural/urban variations [3, 5, 6] just like the findings in this study.

From previous studies, findings demonstrated that children of educated mothers are at lower risk of anaemia [3, 5, 6, 23]. However, our study did not prove this relationship between children’s mother level of education or access to education facilities (e.g. listening to the radio) and anaemia. Likewise, childhood anaemia relationship with pregnancy status of their mothers was not established in this study. On the other hand, this study showed that anaemia in the whole cohort and children between the age of 2 and under 5 years is predicted by maternal anaemia. Such a relationship was documented in previous studies but rather in young children (6 to 23 months) [5, 6].

Relationship between anaemia and economic status as a predictor is well established. Anaemia is more prevalent in low-income countries than in middle-income countries [6, 7]. A family with a large number of household members could compete in available resources including food, but could also contribute to increasing the household income. However, this study and other research [5] found no association between anaemia and household size. Nonetheless, a higher risk of anaemia in children has previously been reported among families with more children [5]. On the other hand, the economic status of the family was identified as a risk factor for anaemia in children under 2 years old, where the poorest were at higher risk of anaemia [3, 5–7]. The same result was obtained in this study for this age group. Why the wealth index effect on anaemia was only on younger children rather than on the older ones is not clear. The status of having health insurance for children was not associated with anaemia as this study demonstrated. However, the role of health insurance could be indirect via reducing the out of pocket expenditure and hence contributing to reducing poverty and its consequences.

Soil-transmitted helminths and schistosomiasis are known causes of anaemia [32, 33]. These infections are linked with access to safe water and sanitation as well as poverty [34, 35]. However, this study did not establish any association between anaemia and access to safe water and sanitation services in Sudan. This may be due to the low and focal prevalence of these infections in Sudan [35].

Fever is a strong indicator of inflammation and infection. Studies conducted in Uganda and Cameroon found that children with a history of fever were at higher risk of being anaemic [26, 36]. This is also supported by an analysis of 52 Demographic Health Surveys in low and low-middle income countries including Africa [5]. Since the present study did not predict such effect of history of fever on anaemia, the possibility of non-infectious causes of anaemia in Sudan needs to be well elaborated.

Malaria is known to cause anaemia through different mechanisms that include a decrease in erythrocytes production or an increase in erythrocytes loss or both [6]. Malaria related anaemia is more prominent in moderate to high transmission areas where individuals are exposed to a high rate of infectious mosquito bites per year [37]. Results of this study demonstrated the link between malaria infection as a predicting factor for anaemia, especially in older children, with children with malaria infection being at higher risk of anaemia. This link is well established [3, 30, 36]. Nonetheless, this association was not evident in under 2 years old children in this study. On the other hand, such a link between anaemia and levels of malaria transmission was not identified in this study. This could be justified within its context that the population of Sudan are living in seasonal low to moderate malaria transmission areas [38] and thus the mechanism of anaemia here could be related to processes related to acute malaria rather than exposure to repeated infections.

## Conclusions

Around half of the children under the age of 5 years in Sudan are anaemic, with worse prevalence in children under 2 years old. Variation in anaemia prevalence between states and in younger and older children was noticed. Different anaemia predictors were identified in the two age groups. While only economic factor was identified as a predictor of anaemia in young children, predictors in older children included the place of residence, maternal anaemia and malaria infection.

As a severe public health problem, the situation of anaemia in Sudan calls for urgent interventions. Efforts targeted at improving socio-economic status, decreasing maternal anaemia and childhood malaria infection may contribute to reducing the magnitude of the problem. Since the problem of childhood anaemia and its risk factors vary with age, the need to have different age categories to address the problem and its interventions might be of value.

## Supplementary Information

**Additional file 1.**

**Additional file 2.**

## Data Availability

Original data used in this study is available from the Communicable and Non-communicable diseases Control Directorate, Federal Ministry of Health, Sudan on reasonable request. The director of the Communicable and Non-communicable diseases Control Directorate should be contacted asking for data access, if any.

## Abbreviations

WHO: World Health Organization
IQ: Intelligence quotient
DALYs: Disability-Adjusted Life Year
IDPs: Internally displaced persons
RDT: Malaria rapid diagnostic test
PCA: Principal component analysis
95%CI: 95% confidence intervals
SD: Standard deviation
OR: Odds ratio
