# Supplementary material for: Anaemia prevalence and determinants in under 5 years children: findings of a cross-sectional population-based study in Sudan
Source: BMC Pediatr. 2020 Nov 30;20:538. doi: 10.1186/s12887-020-02434-w (PMC7702668; doi:10.1186/s12887-020-02434-w)
Supplement: Supplementary file 1 — Additional file 1. [file 12887_2020_2434_MOESM1_ESM.docx]

Sudan Malaria Indicator Survey - 2016

# Household Members Questionnaire

## October 2016

**FEDERAL MINISTRY OF HEALTH**

**SUDAN MALARIA INDICATOR SURVEY - 2016**

**HOUSEHOLD MEMBERS QUESTIONNAIRE**

| **QUESTIONNAIRE ID [___] [___]-[___] [___]-[___] [___] TO BE SUMMARIZED BY TEAM SUPERVISOR**  **State Cluster Household** |
| --- |
| STATE NAME……………………………………………………………………………………………………………………………………………….  STATE CODE [_____] [_____]  LOCALITY NAME…………………………………………………………………………………………………………………………………  LOCALITY CODE [_____] [_____]  CLUSTER NAME…………………………………………………………………………………………………………………………………..    CLUSTER CODE [_____] [_____]  HOUSEHOLD CODE [_____] [_____]  NAME OF HOUSEHOLD HEAD …………………………………………………………………………………………………………….  URBAN / RURAL / IDP (URBAN=1, RURAL=2, IDP camp=3) [_____] |
|  |

| **INTERVIEWER VISITS** | | | | | |
| --- | --- | --- | --- | --- | --- |
|  | 1 | 2 | 3 | FINAL VISIT | |
| DATE  INTERVIEWER’S NAME  RESULT***** |  |  |  | ┌──┬──┐  DAY │░░│░░│  ├──┼──┤  MONTH │░░│░░│  ┌──┬──┼──┼──┤  YEAR │░░│ │░░│░░│  └──┴──┼──┼──┤  NAME │░░│░░│  └──┼──┤  RESULT │░░│  └──┘ | |
| NEXT VISIT: DATE |  |  |  | TOTAL NO. OF VISITS | ┌──┐  │░░│  └──┘ |
| TIME |  |  |  |  |  |
| ***RESULT CODES**:  1 COMPLETED  2 NOT AT HOME  3 POSTPONED | 4 REFUSED  5 PARTLY COMPLETED  6 INCAPACITATED | | 7 OTHER __________________________  (SPECIFY) | | |

COUNTRY-SPECIFIC INFORMATION: LANGUAGE OF QUESTIONNAIRE, LANGUAGE OF INTERVIEW, NATIVE LANGUAGE OF RESPONDENT, AND WHETHER TRANSLATOR USED

| FIELD SUPERVISOR | | STATE COORDINATOR | NATIONAL COORDINATOR | DATE ENTRY |
| --- | --- | --- | --- | --- |
| NAME | ┌──┬──┐  │░░│░░│  └──┴──┘ | ┌──┬──┐  │░░│░░│  └──┴──┘ | ┌──┬──┐  │░░│░░│ | ┌──┬──┐  │░░│░░│  └──┴──┘ |
| DATE |  |  | └──┴──┘ |  |

**INTRODUCTION AND CONSENT**

| **INFORMED CONSENT (HOUSEHOLD MEMBER 1)**  Hello. My name is and I am working with National Malaria Control Programme, Federal Ministry of Health. We are conducting a national survey about malaria. We would very much appreciate your participation in this survey. The information you provide will help the government to plan malaria services. The survey usually takes between 10 and 20 minutes to complete. Whatever information you provide will be kept strictly confidential and will not be shown to other persons. As part of this survey, we are studying malaria among all persons. Malaria is a serious health problem that results from the bite of an infected mosquito. This survey will assist the government to develop programs to prevent and treat this important health problem.  Participation in this survey is voluntary and you can choose not to answer any individual question or all of the questions. However, we hope that you will participate in this survey since your views are important.  We request that everyone in the household give a few drops of blood from a finger for malaria testing. The test uses disposable sterile instruments that are clean and completely safe. The results of malaria and hemoglobin testing will be given to you right after the blood is taken. The results will be kept confidential. Anyone found to have malaria will be treated straight away by with nationally recommended medicines. Severe cases or cases that needs special care will be referred to the public health facility / services.  May I now ask that (NAME OF PERSON]) participate in the malaria test. However, if you decide not to have you or your child tested, it is your right and we will respect your decision.  At this time, do you want to ask me anything about the survey?  May I begin the interview now and then do the blood test afterwards?  Signature of interviewee: Date: | |
| --- | --- |
| RESPONDENT AGREES TO BE INTERVIEWED 1  🡫 | RESPONDENT DOES NOT AGREE TO BE INTERVIEWED 2 ──END |

| **INFORMED CONSENT (HOUSEHOLD MEMBER 2)**  Hello. My name is and I am working with National Malaria Control Programme, Federal Ministry of Health. We are conducting a national survey about malaria. We would very much appreciate your participation in this survey. The information you provide will help the government to plan malaria services. The survey usually takes between 10 and 20 minutes to complete. Whatever information you provide will be kept strictly confidential and will not be shown to other persons. As part of this survey, we are studying malaria among all persons. Malaria is a serious health problem that results from the bite of an infected mosquito. This survey will assist the government to develop programs to prevent and treat this important health problem.  Participation in this survey is voluntary and you can choose not to answer any individual question or all of the questions. However, we hope that you will participate in this survey since your views are important.  We request that everyone in the household give a few drops of blood from a finger for malaria testing. The test uses disposable sterile instruments that are clean and completely safe. The results of malaria and hemoglobin testing will be given to you right after the blood is taken. The results will be kept confidential. Anyone found to have malaria will be treated straight away by with nationally recommended medicines. Severe cases or cases that needs special care will be referred to the public health facility / services.  May I now ask that (NAME OF PERSON]) participate in the malaria test. However, if you decide not to have you or your child tested, it is your right and we will respect your decision.  At this time, do you want to ask me anything about the survey?  May I begin the interview now and then do the blood test afterwards?  Signature of interviewee: Date: | |
| --- | --- |
| RESPONDENT AGREES TO BE INTERVIEWED 1  🡫 | RESPONDENT DOES NOT AGREE TO BE INTERVIEWED 2 ──END |

**SECTION 2A**

|  | ENTER IN THE TABLE THE LINE NUMBER AND NAME OF EACH INDIVIDUAL (Use additional pages for households with more than two persons).  **Begin by ‘now I would like to ask you some questions about your health** ‘. | | |
| --- | --- | --- | --- |
| 201 | NAME AND LINE NUMBER  FROM pages 2 and 3 of the Household Questionnaires | ┌──┬──┐  LINE NUMBER │░░│░░│  └──┴──┘  NAME | ┌──┬──┐  LINE NUMBER │░░│░░│  └──┴──┘  NAME |
| 202 | IF CHILD **LESS THAN FIVE YEARS** TYPE NAME AND LINE NUMBER OF PERSON WHO RESPONDED TO HIS QUESTIONS (HIS/HER MOTHER OR GUARDIAN)  FROM pages 2 and 3 of the Household Questionnaires. IF NOT LESS THAN 5 YEARS SKIP TO 203 | MOTHER/GUARDIAN ┌──┬──┐  LINE NUMBER │░░│░░│  └──┴──┘  MOTHER GUARDIAN  NAME | MOTHER/GUARDIAN ┌──┬──┐  LINE NUMBER │░░│░░│  └──┴──┘  MOTHER GUARDIAN  NAME |
| 203 | What is YOUR level of education?  For children under the age of five years record the education level of the mother or guardian! | NO FORMAL EDUCATION………11  RELIGIOUS (KHALWA OR OTHERS)………………………….12  PRIMARY/INTERMEDIATE/BASIC  LEVEL:  PARTIAL ………..……..21  COMPLETE …………..22  SECONDARY:  PARTIAL……………….31  COMPLETE …………..32  ABOVE ……………………………41 | NO FORMAL EDUCATION………11  RELIGIOUS (KHALWA OR OTHERS)………………………….12  PRIMARY/INTERMEDIATE/BASIC  LEVEL:  PARTIAL ………..……..21  COMPLETE …………..22  SECONDARY:  PARTIAL……………….31  COMPLETE …………..32  ABOVE ……………………………41 |
| 204 | Have you been ill with a fever at any time in the last 2 weeks? | YES 1  NO 2  DON’T KNOW..................................8  (If NO OR DON’T KNOW GO TO 215) | YES 1  NO 2  DON’T KNOW..................................8  (If NO OR DON’T KNOW GO TO 215) |
| 205 | For how many days did you have the fever start?  IF LESS THAN ONE DAY, RECORD ‘00’. | ┌────┬────┐  DAYS │░░░░│░░░░│  └────┴────┘  DON’T KNOW………………………98 | ┌────┬────┐  DAYS │░░░░│░░░░│  └────┴────┘  DON’T KNOW………………………98 |
| 206 | Did you seek advice or treatment for the fever from any source? | YES 1  NO 2  (IF NO SKIP TO 215) | YES 1  NO 2  (IF NO SKIP TO 215) |

| 207 | Where did you seek advice or treatment for this fever? CIRCLE FIRST RESPONSE ONLY | PUBLIC SECTOR:  GOVT. HOSPITAL........................A  GOVT. HEALTH CENTER............B  GOVT. BASIC HEALTH UNIT.............................................C  MOBILE CLINIC........................... D  COMMUNITY HEALTH WORKER.....................................E  OTHER PUBLIC G  (SPECIFY)  NGO…………………………………..H  PRIVATE MEDICAL SECTOR:  PRIVATE. HOSPITAL / CLINIC..........................................I  OTHER PRIVATE  MEDICAL J  (SPECIFY)  LOCAL SOURCE:  SHOP/MARKET............................K  TRAD. PRACTITIONER............... L  OTHER SOURCE:  PHARMACY / DRUG STORE…………………................M  OTHER X  (SPECIFY) | PUBLIC SECTOR:  GOVT. HOSPITAL........................A  GOVT. HEALTH CENTER............B  GOVT. BASIC HEALTH UNIT.............................................C  MOBILE CLINIC........................... D  COMMUNITY HEALTH WORKER.....................................E  OTHER PUBLIC G  (SPECIFY)  NGO…………………………………..H  PRIVATE MEDICAL SECTOR:  PRIVATE. HOSPITAL / CLINIC..........................................I  OTHER PRIVATE  MEDICAL J  (SPECIFY)  LOCAL SOURCE:  SHOP/MARKET............................K  TRAD. PRACTITIONER............... L  OTHER SOURCE:  PHARMACY / DRUG STORE…………………................M  OTHER X  (SPECIFY) |  |
| --- | --- | --- | --- | --- |
| 208 | How long did it take you to get to the place of care OR source of treatment? | [____] [____] [____] minutes | [____] [____] [____] minutes |  |
| 209 | How many days after the fever began did you first seek treatment for fever?  IF THE SAME DAY, RECORD ‘00’. | ┌────┬────┐  DAYS │░░░░│░░░░│  └────┴────┘ | ┌────┬────┐  DAYS │░░░░│░░░░│  └────┴────┘ |  |
| 210 | Was a blood test for malaria done on you? | YES ...................................................1  NO ....................................................2  DON’T KNOW..................................8  If NO or DON’T KNOW Go to 215 | YES ...................................................1  NO ....................................................2  DON’T KNOW..................................8  If NO or DON’T KNOW Go to 215 |  |
| 211 | Was a Rapid Diagnostic Test or Blood Smear used?  (show respondent example of slide and RDT) | Rapid Diagnostic Test……..………..1  Blood Smear………………..………..2  Both…………………………...………3  Don’t know…………………………….4 | Rapid Diagnostic Test……..……….1  Blood Smear………………..……….2  Both…………………………...……..3  Don’t know…………………………….4 |  |
| 212 | Was your results POSITIVE for malaria? (any one if you did both) | POSITIVE.........................................1  NEGATIVE.......................................2  NOT TOLD.......................................3  DON’T KNOW..................................8 | POSITIVE.........................................1  NEGATIVE.......................................2NOT TOLD.......................................3  DON’T KNOW..................................8 |  |
| 213 | At any time during the illness, did you take any drugs for the fever? | YES ...................................................1  NO.................................................... 2  DON’T KNOW.................................. 8  (IF NO OR DON’T KNOW SKIP TO 215) | YES ...................................................1  NO .....................................................2  DON’T KNOW...................................8  (IF NO OR DON’T KNOW SKIP TO 215) |  |

| 214 | What drugs did you take?^1^  Any other drugs?  RECORD ALL MENTIONED. | ANTIMALARIAL  ACT (AS+SP) …………....……….A  ACT (AL)…………………………..B  SP/FANSIDAR C  CHLOROQUINE D  AMODIAQUINE + AS E  QUININE F  ARTHEMETER……………………G  OTHER  ANTIMALARIAL H  (SPECIFY)  ANTIPYRETICS…………………...…..I  OTHER _ X  (SPECIFY)  DON’T KNOW Z | ANTIMALARIAL  ACT (AS+SP) …………....……….A  ACT (AL)…………………………..B  SP/FANSIDAR C  CHLOROQUINE D  AMODIAQUINE + AS E  QUININE F  ARTHEMETER……………………G  OTHER  ANTIMALARIAL H  (SPECIFY)  ANTIPYRETICS..……………………..I  OTHER _ X  (SPECIFY)  DON’T KNOW Z |
| --- | --- | --- | --- |
| 215 | Did you sleep under a bed net last night? | 1. YES 2. NO | 1. YES 2. NO |
| 216 | If YES to 215, which type of bed net did you sleep under last night? | 1. LLIN 2. NOT TREATED NET 3. OTHER TYPE OF NET (LOCALY / HOME MADE) | 1. LLIN 2. NOT TREATED NET 3. OTHER TYPE OF NET (LOCALY / HOME MADE) |
| 217 | If NO to 215, why did you not sleep under a mosquito net? | 1. WE DON’T HAVE NETS IN THE HOUSEHOLD 2. WE DON’T HAVE ENOUGH NETS IN THE HOUSEHOLD 3. WE HAVE NETS BUT DON’T HAVE THE SPACE TO HANG THE NETS IN THE HOUSEHOLD 4. TOO HOT TO SLEEP UNDER A NET 5. NO MOSQUITOES AT THE MOMENT 6. WE DON’T HAVE MALARIA HERE 7. DON’T KNOW HOW TO HANGE THE NET 8. DON’T BELIEVE THE NET IS USEFULL 9. HANGING THE NET ON THE BED BOTHERING ME | 1. WE DON’T HAVE NETS IN THE HOUSEHOLD 2. WE DON’T HAVE ENOUGH NETS IN THE HOUSEHOLD 3. WE HAVE NETS BUT DON’T HAVE THE SPACE TO HANG THE NETS IN THE HOUSEHOLD 4. TOO HOT TO SLEEP UNDER A NET 5. NO MOSQUITOES AT THE MOMENT 6. WE DON’T HAVE MALARIA HERE 7. DON’T KNOW HOW TO HANGE THE NET 8. DON’T BELIEVE THE NET IS USEFULL 9. HANGING THE NET ON THE BED BOTHERING ME |
| 218 | Is (NMAE) covered with health insurance? | 1. YES 2. NO   If NO Go for the malaria and hemoglobin testing SECTION 2B | 1. YES 2. NO   If NO Go for the malaria and hemoglobin testing SECTION 2B |
| 219 | If yes to 218 above, what kind of insurance facility? | 1. Public health insurance 2. Police health insurance 3. Military health insurance 4. Private health insurance | 1. Public health insurance 2. Police 3. Military 4. Private health insurance |
| 220 | Does the (NAME) has Diabetes Mellitus? | 1. YES 2. NO | 1. YES 2. NO |
| 221 | Does the (NAME) has Hypertension? | 1. YES 2. NO | 1. YES 2. NO |
| 222 | Has the NAME been ill during the last 3 months? | 1. YES 2. NO   If NO skip to 235 | 1. YES 2. NO   If NO skip to 235 |
| 222a | If yes to 220 above, has the NAME used any kind of treatment before going to health facility? | 1. YES 2. NO   If NO skip to 225 | 1. YES 2. NO   If NO skip to 225 |
| 222b | If yes to 220 above, describe the type of treatment used | 1. Medical drugs 2. Traditional 3. Both | 1. Medical drugs 2. Traditional 3. Both |
| 223 | What was the cost of the medical drugs? IN SUDANESE POUNDS. WRITE 00 IF NOTHING | SDG [___] [___][___] [___][___] | SDG [___] [___][___] [___][___] |
| 224 | What was the cost of the traditional treatment? IN SUDANESE POUNDS. WRITE 00 IF NOTHING | SDG [___] [___][___] [___][___] | SDG [___] [___][___] [___][___] |
| 225 | Was the NAME able to perform his routine job/work during his illness? | 1. YES 2. NO   If NO skip to 227 | 1. YES  2. NO  If NO skip to 227 |
| 226 | If yes to 225 above, for how many days the NAME was unable to perform his/her routine job/work? | [___][___] days | [___][___] days |
| 227 | How many times the NAME visited a health facility for this illness in the last 3 months? IF NON WRITE 00 | [___][___] times | [___][___] times |
| 228 | How much was the total cost for this illness including all number of visits? IN SUDANESE POUNDS. WRITE 00 IF NOTHING | SDG [___] [___][___] [___][___] | SDG [___] [___][___] [___][___] |
| 229 | How much was the cost of travel of the ill person to the health facility in the last visit? IN SUDANESE POUNDS. WRITE 00 IF NOTHING | SDG [___] [___][___] [___][___] | SDG [___] [___][___] [___][___] |
| 230 | How much was the cost of travel of the co-patient to the health facility in the last visit? IN SUDANESE POUNDS. WRITE 00 IF NOTHING | SDG [___] [___][___] [___][___] | SDG [___] [___][___] [___][___] |
| 231 | How much was the cost of consultation / using the health facility service in the last visit? IN SUDANESE POUNDS. WRITE 00 IF NOTHING | SDG [___] [___][___] [___][___] | SDG [___] [___][___] [___][___] |
| 232 | How much was the cost of investigation in the last visit? IN SUDANESE POUNDS. WRITE 00 IF NOTHING | SDG [___] [___][___] [___][___] | SDG [___] [___][___] [___][___] |
| 233 | How much was the cost of treatment in the last visit? IN SUDANESE POUNDS. WRITE 00 IF NOTHING | SDG [___] [___][___] [___][___] | SDG [___] [___][___] [___][___] |
| 234 | If not sure about the details above about (cost of consultation, diagnosis, treatment, travel etc.), how much was the overall cost for this illness for this last visit only? IN SUDANESE POUNDS. WRITE 00 IF NOTHING | SDG [___] [___][___] [___][___] | SDG [___] [___][___] [___][___] |
| 235 | Has the NAME been admitted to a hospital during the last 6 months? | - - - 1. YES       2. NO   If NO skip to 239 | 1. YES 2. NO   If NO skip to 239 |
| 236 | If YES to 235 above, how much was the overall cost (cost of consultation, diagnosis, scans, admission, treatment, co-patient, travel etc.), for this admission? IN SUDANESE POUNDS. WRITE 00 IF NOTHING | SDG [___] [___][___] [___][___] | SDG [___] [___][___] [___][___] |
| 237 | Has anyone or institute participated in the cost of this admission? | 1. YES 2. NO   If NO skip to 239 | 1. YES 2. NO   If NO skip to 239 |
| 238 | If yes to 237 above, mention who and how much cost he shared? | 1. Federal Government (not Zakat or working institution Employee)   SDG [___] [___][___] [___][___] | 1. Federal Government (not Zakat or working institution Employee)   SDG [___] [___][___] [___][___] |
|  |  | 1. State Government (not Zakat or working institution Employee)   SDG [___] [___][___] [___][___] | 1. State Government (not Zakat or working institution Employee)   SDG [___] [___][___] [___][___] |
|  |  | 1. Private company (not Zakat)   SDG [___] [___][___] [___][___] | 1. Private company (not Zakat)   SDG [___] [___][___] [___][___] |
|  |  | 1. Health Insurance   SDG [___] [___][___] [___][___] | 1. Health Insurance   SDG [___] [___][___] [___][___] |
|  |  | 1. Zakat   SDG [___] [___][___] [___][___] | 1. Zakat   SDG [___] [___][___] [___][___] |
|  |  | 1. working institution Employee   SDG [___] [___][___] [___][___] | 1. working institution Employee   SDG [___] [___][___] [___][___] |
|  |  | 1. Relatives, friends, charity, etc   SDG [___] [___][___] [___][___] | 1. Relatives, friends, charity, etc   SDG [___] [___][___] [___][___] |
| 239 | Has the expenditure on health incurred any burden on the household income during the last 12 months? | 1. YES 2. NO | 1. YES 2. NO |
| 240 | If YES to 239 above, how did you cope with this? | 1. bout some medicines and left others | 1. bout some medicines and left others |
|  |  | 1. additional work / overtime | 1. additional work / overtime |
|  |  | 1. reduced expenditure on other household items | 1. reduced expenditure on other household items |
|  |  | 1. used household savings | 1. used household savings |
|  |  | 1. sold some household items | 1. sold some household items |
|  |  | 1. borrow money from someone | 1. borrow money from someone |
|  |  | 1. assistance from someone/charity | 1. assistance from someone/charity |
|  |  | 1. support from relatives, friends, etc from Sudan or outside | 1. support from relatives, friends, etc from Sudan or outside |
|  |  | 1. others specify ______________ | 1. others specify ______________ |

**SECTION 2B: MALARIA AND HEMOGLOBIN TESTING**

CHECK COLUMN (7) OF HOUSEHOLD LISTING: RECORD THE LINE NUMBER, NAME AND AGE OF PERSON TO BE TESTED FOR PARASITES. **AFTER COLLECTING SAMPLE FOR RDT, PLEASE USE THIS FINGER PRICK BLOOD FOR ALSO MEASURING Hb. AND SPOTTING THE FILTER PAPER TO AVOID MULTIPLE PRICKING FOR INDIVIDUAL.**

| **Malaria and Hb. TESTING** | | | | | |
| --- | --- | --- | --- | --- | --- |
| 241.LINE NUMBER  From HOUSEHOLD LISTING in HOUSEHOLD QUESTIONNAIRE | 242.NAME From HOUSEHOLD LISTING in HOUSEHOLD QUESTIONNAIRE | 243. Are you sick with fever today? | 244. INDICATE IF PARTCIPANT AGREED TO MALARIA TEST.  (CIRCLE CODE) | 245.RDT RESULT | 246.Hb RESULT CHILDREN 6 MONTHS TO LESS THAN 5 YEARS and WOMEN IN THE REPRODUCTIVE AGE |
|  |  |  | CONSENT GRANTED / CONSENT REFUSED | Enter code 1 to 7  1= *P.f.* positive  2= *P.v.* positive  3= mix positive  4= negative,  5=invalid,  6=not done, parent/guardian refused,  7- not done, patient refused. | Enter Hb level [Hemocue^TM^ reading] (g/dl): |
| LINE NUMBER  ┌──┬──┐  │░░│░░│  └──┴──┘ | NAME  _______________ | sick with fever today:  1 = YES  2= NO | CONSENT GRANTED = 1  CONSENT REFUSED = 2 | RDT RESULT  [____] | Is the NAME a CHILDREN 6 MONTHS TO LESS THAN 5 YEARS or a WOMEN AGED 15 - 49 YEARS?  YES = 1 NO = 2  If yes record the Hb. RESULT  [___] [___]. [___] g/dl |
| LINE NUMBER  ┌──┬──┐  │░░│░░│  └──┴──┘ | NAME  _______________ | sick with fever today:  1 = YES  2= NO | CONSENT GRANTED = 1  CONSENT REFUSED = 2 | RDT RESULT  [____] | Is the NAME a CHILDREN 6 MONTHS TO LESS THAN 5 YEARS or a WOMEN AGED 15 - 49 YEARS?  YES = 1 NO = 2  If yes record the Hb. RESULT  [___] [___]. [___] g/dl |
| **FILTER PAPERS** | | | | | |
| **TICK HERE IF:** FILTER PAPERS DONE AND LABELLED FOR PERSON 1 □ FILTER PAPERS DONE AND LABELLED FOR PERSON 2 □ | | | | | |

**Please inform all individuals of their test status. Where individuals have tested positive please offer them appropriate treatment. They are at liberty to accept or decline testing or treatment.**

INTERVIEWER’S OBSERVATIONS

TO BE FILLED IN AFTER COMPLETING INTERVIEW

COMMENTS ABOUT RESPONDENT:

|  |
| --- |
|  |
|  |
|  |

COMMENTS ON SPECIFIC QUESTIONS:

|  |
| --- |
|  |
|  |
|  |

ANY OTHER COMMENTS:

|  |
| --- |
|  |
|  |
|  |

SUPERVISOR’S OBSERVATIONS

|  |
| --- |
|  |
|  |
|  |
|  |
|  |

NAME OF THE SUPERVISOR:______________________________________ DATE: __________________________
