# Supplementary material for: Anaemia prevalence and determinants in under 5 years children: findings of a cross-sectional population-based study in Sudan
Source: BMC Pediatr. 2020 Nov 30;20:538. doi: 10.1186/s12887-020-02434-w (PMC7702668; doi:10.1186/s12887-020-02434-w)
Supplement: Supplementary file 2 — Additional file 2. [file 12887_2020_2434_MOESM2_ESM.docx]

Sudan Malaria Indicator Survey - 2016

# Household Questionnaire

**October 2016**

**FEDERAL MINISTRY OF HEALTH**

**SUDAN MALARIA INDICATOR SURVEY - 2016**

**HOUSEHOLD QUESTIONNAIRE**

| **QUESTIONNAIRE ID [___] [___]-[___] [___]-[___] [___] TO BE SUMMARIZED BY TEAM SUPERVISOR**  **S C H** | | | | | |
| --- | --- | --- | --- | --- | --- |
| STATE NAME……………………………………………………………………………………………………………………………………………….  STATE CODE [_____] [_____]  LOCALITY NAME…………………………………………………………………………………………………………………………………………  LOCALITY CODE [_____] [_____]  CLUSTER NAME…………………………………………………………………………………………………………………………………………..    CLUSTER CODE [_____] [_____]  HOUSEHOLD CODE [_____] [_____]  NAME OF HOUSEHOLD HEAD………………………………………………………………………………………………………………………….  URBAN / RURAL / IDP (URBAN=1, RURAL=2, IDP camp=3) [_____] | | | | | |
| GPS READINGS: LATITUDE: [__ __ __] **.** [__ __ __ __ __ ] LONGITUDE: [__ __ __] **.** [__ __ __ __ __ ] | | | | | |
| **INTERVIEWER VISITS** | | | | | |
|  | 1 | 2 | 3 | FINAL VISIT | |
| DATE  INTERVIEWER’S NAME  RESULT***** |  |  |  | ┌──┬──┐  DAY │░░│░░│  ├──┼──┤  MONTH │░░│░░│  ┌──┬──┼──┼──┤  YEAR │░░│ │░░│░░│  └──┴──┼──┼──┤  NAME │░░│░░│  └──┼──┤  RESULT │░░│  └──┘ | |
| NEXT VISIT: DATE |  |  |  | TOTAL NO. OF VISITS | ┌──┐  │░░│  └──┘ |
| TIME |  |  |  |  |  |
| ***** **RESULT CODES**:  1 COMPLETED  2 NO HOUSEHOLD MEMBER AT HOME OR NO COMPETENT RESPONDENT AT HOME AT TIME OF VISIT  3 ENTIRE HOUSEHOLD ABSENT FOR EXTENDED PERIOD OF TIME  4 POSTPONED  5 REFUSED  6 DWELLING VACANT OR ADDRESS NOT A DWELLING  7 DWELLING DESTROYED  8 DWELLING NOT FOUND  9 OTHER  (SPECIFY) | | | | TOTAL  PERSONS IN HOUSEHOLD | ┌──┬──┐  │░░│░░│  └──┴──┘ |
|  |  |  |  | TOTAL ELIGIBLE WOMEN | ┌──┬──┐  │░░│░░│  └──┴──┘ |
|  |  |  |  | LINE NUMBER OF RESPONDENT TO HOUSEHOLD QUESTIONNAIRE | ┌──┬──┐  │░░│░░│  └──┴──┘ |

| FIELD SUPERVISOR | | STATE COORDINATOR | NATIONAL COORDINATOR | DATA ENTRY |
| --- | --- | --- | --- | --- |
| NAME___________________ |  |  |  |  |
|  |  |  |  |  |

**HOUSEHOLD LISTING**

Now we would like some information about the people who usually live in your household or who are staying with you now.

| LINE NO. | USUAL RESIDENTS AND VISITORS | RELATIONSHIP TO HEAD OF HOUSEHOLD | SEX | RESIDENCE | | AGE | | AGE | | ELIGIBLE WOMEN | |
| --- | --- | --- | --- | --- | --- | --- | --- | --- | --- | --- | --- |
|  | Please give me the names of the persons who usually live in your household and guests of the household who stayed here last night, starting with the head of the household. | What is the relationship of (NAME) to the head of the household?***** | Is (NAME) male or female? | Does (NAME) usually live here? | Did (NAME) stay here last night? | How old is (NAME)?  Write 00 for children less than 12 months | | If (NAME) old is less than 12 months, write the exact age in months | | CIRCLE LINE NUMBER OF ALL WOMEN AGE 15‑49 WHO ARE MARRIED NOW OR HAVE BEEN MARRIED BEFORE | Are you (woman 15-49 years old) PREGNANT? |
| (1) | (2) | (3) | (4) | (5) | (6) | (7) | | 7 A | | (8) | 8 A |
|  |  |  | M F | YES NO | YES NO | IN YEARS | | IN MONTHS | |  | YES NO |
| 01 |  | ┌──┬──┐  │░░│░░│  └──┴──┘ | 1 2 | 1 2 | 1 2 | ┌──┬──┐  │░░│░░│  └──┴──┘ | | ┌──┬──┐  │░░│░░│  └──┴──┘ | | 01 | 1 2 |
| 02 |  | ┌──┬──┐  │░░│░░│  └──┴──┘ | 1 2 | 1 2 | 1 2 | ┌──┬──┐  │░░│░░│  └──┴──┘ | | ┌──┬──┐  │░░│░░│  └──┴──┘ | | 02 | 1 2 |
| 03 |  | ┌──┬──┐  │░░│░░│  └──┴──┘ | 1 2 | 1 2 | 1 2 | ┌──┬──┐  │░░│░░│  └──┴──┘ | | ┌──┬──┐  │░░│░░│  └──┴──┘ | | 03 | 1 2 |
| 04 |  | ┌──┬──┐  │░░│░░│  └──┴──┘ | 1 2 | 1 2 | 1 2 | ┌──┬──┐  │░░│░░│  └──┴──┘ | | ┌──┬──┐  │░░│░░│  └──┴──┘ | | 04 | 1 2 |
| 05 |  | ┌──┬──┐  │░░│░░│  └──┴──┘ | 1 2 | 1 2 | 1 2 | ┌──┬──┐  │░░│░░│  └──┴──┘ | | ┌──┬──┐  │░░│░░│  └──┴──┘ | | 05 | 1 2 |
| 06 |  | ┌──┬──┐  │░░│░░│  └──┴──┘ | 1 2 | 1 2 | 1 2 | ┌──┬──┐  │░░│░░│  └──┴──┘ | | ┌──┬──┐  │░░│░░│  └──┴──┘ | | 06 | 1 2 |
| 07 |  | ┌──┬──┐  │░░│░░│  └──┴──┘ | 1 2 | 1 2 | 1 2 | ┌──┬──┐  │░░│░░│  └──┴──┘ | | ┌──┬──┐  │░░│░░│  └──┴──┘ | | 07 | 1 2 |
| 08 |  | ┌──┬──┐  │░░│░░│  └──┴──┘ | 1 2 | 1 2 | 1 2 | ┌──┬──┐  │░░│░░│  └──┴──┘ | | ┌──┬──┐  │░░│░░│  └──┴──┘ | | 08 | 1 2 |
| 09 |  | ┌──┬──┐  │░░│░░│  └──┴──┘ | 1 2 | 1 2 | 1 2 | ┌──┬──┐  │░░│░░│  └──┴──┘ | | ┌──┬──┐  │░░│░░│  └──┴──┘ | | 09 | 1 2 |
| 10 |  | ┌──┬──┐  │░░│░░│  └──┴──┘ | 1 2 | 1 2 | 1 2 | ┌──┬──┐  │░░│░░│  └──┴──┘ | | ┌──┬──┐  │░░│░░│  └──┴──┘ | | 10 | 1 2 |
| *** CODES FOR Q.3**  RELATIONSHIP TO HEAD OF HOUSEHOLD:  01 = HEAD  02 = WIFE/HUSBAND  03 = SON OR  DAUGHTER  04 = SON-IN-LAW OR  DAUGHTER-IN-LAW  05 = GRANDCHILD | | 06 = PARENT  07 = PARENT-IN-LAW  08 = BROTHER OR SISTER  09 = OTHER RELATIVE  10 = ADOPTED/FOSTER/  STEPCHILD  11 = ETHIOPIAN or ARTRIAN WORKER  12 = NOT RELATED  98 = DON’T KNOW | |  | | |  | |  |  |  |

| LINE NO. | USUAL RESIDENTS AND VISITORS | RELATIONSHIP TO HEAD OF HOUSEHOLD | SEX | RESIDENCE | | AGE | AGE (in months) | ELIGIBLE WOMEN | |
| --- | --- | --- | --- | --- | --- | --- | --- | --- | --- |
|  | Please give me the names of the persons who usually live in your household and guests of the household who stayed here last night, starting with the head of the household. | What is the relationship of (NAME) to the head of the household?***** | Is (NAME) male or female? | Does (NAME) usually live here? | Did (NAME) stay here last night? | How old is (NAME)?  Write 00 for children less than 12 months  Write 99 if age is 100 or above | If (NAME) old is less than 12 months, write the exact age in months bellow | CIRCLE LINE NUMBER OF ALL WOMEN AGE 15‑49 WHO ARE MARRIED NOW OR HAVE BEEN MARRIED BEFORE | Are you (woman 15-49 years old) PREGNANT? |
| (1) | (2) | (3) | (4) | (5) | (6) | (7) | 7 A | (8) | 8 A |
|  |  |  | M F | YES NO | YES NO | IN YEARS | IN MONTHS |  | YES NO |
| 11 |  | ┌──┬──┐  │░░│░░│  └──┴──┘ | 1 2 | 1 2 | 1 2 | ┌──┬──┐  │░░│░░│  └──┴──┘ | ┌──┬──┐  │░░│░░│  └──┴──┘ | 11 | 1 2 |
| 12 |  | ┌──┬──┐  │░░│░░│  └──┴──┘ | 1 2 | 1 2 | 1 2 | ┌──┬──┐  │░░│░░│  └──┴──┘ | ┌──┬──┐  │░░│░░│  └──┴──┘ | 12 | 1 2 |
| 13 |  | ┌──┬──┐  │░░│░░│  └──┴──┘ | 1 2 | 1 2 | 1 2 | ┌──┬──┐  │░░│░░│  └──┴──┘ | ┌──┬──┐  │░░│░░│  └──┴──┘ | 13 | 1 2 |
| 14 |  | ┌──┬──┐  │░░│░░│  └──┴──┘ | 1 2 | 1 2 | 1 2 | ┌──┬──┐  │░░│░░│  └──┴──┘ | ┌──┬──┐  │░░│░░│  └──┴──┘ | 14 | 1 2 |
| 15 |  | ┌──┬──┐  │░░│░░│  └──┴──┘ | 1 2 | 1 2 | 1 2 | ┌──┬──┐  │░░│░░│  └──┴──┘ | ┌──┬──┐  │░░│░░│  └──┴──┘ | 15 | 1 2 |
| 16 |  | ┌──┬──┐  │░░│░░│  └──┴──┘ | 1 2 | 1 2 | 1 2 | ┌──┬──┐  │░░│░░│  └──┴──┘ | ┌──┬──┐  │░░│░░│  └──┴──┘ | 16 | 1 2 |
| 17 |  | ┌──┬──┐  │░░│░░│  └──┴──┘ | 1 2 | 1 2 | 1 2 | ┌──┬──┐  │░░│░░│  └──┴──┘ | ┌──┬──┐  │░░│░░│  └──┴──┘ | 17 | 1 2 |
| 18 |  | ┌──┬──┐  │░░│░░│  └──┴──┘ | 1 2 | 1 2 | 1 2 | ┌──┬──┐  │░░│░░│  └──┴──┘ | ┌──┬──┐  │░░│░░│  └──┴──┘ | 18 | 1 2 |
| 19 |  | ┌──┬──┐  │░░│░░│  └──┴──┘ | 1 2 | 1 2 | 1 2 | ┌──┬──┐  │░░│░░│  └──┴──┘ | ┌──┬──┐  │░░│░░│  └──┴──┘ | 19 | 1 2 |
| 20 |  | ┌──┬──┐  │░░│░░│  └──┴──┘ | 1 2 | 1 2 | 1 2 | ┌──┬──┐  │░░│░░│  └──┴──┘ | ┌──┬──┐  │░░│░░│  └──┴──┘ | 20 | 1 2 |

| TICK HERE IF CONTINUATION SHEET USED | | ┌──┐  └──┘ | | | | | |
| --- | --- | --- | --- | --- | --- | --- | --- |
| **Just to make sure that I have a complete listing:** | | | | | | | |
| 1) | Are there any other persons such as small children or infants that we have not listed? | | YES | ┌──┐  └──┴─> | ENTER EACH IN TABLE | NO | ┌──┐  └──┘ |
| 2) | Are there anyone at school today that might have been missed that we have not listed? | | YES | ┌──┐  └──┴─> | ENTER EACH IN TABLE | NO | ┌──┐  └──┘ |
| 3) | In addition, are there any other people who may not be members of your family, such as domestic servants, lodgers or friends who usually live here? | | YES | ┌──┐  └──┴─> | ENTER EACH IN TABLE | NO | ┌──┐  └──┘ |
| 4) | Are there any guests or temporary visitors staying here, or anyone else who stayed here last night, who have not been listed? | | YES | ┌──┐  └──┴─> | ENTER EACH IN TABLE | NO | ┌──┐  └──┘ |

| NO. | QUESTIONS AND FILTERS | CODING CATEGORIES | SKIP | |
| --- | --- | --- | --- | --- |
| 9 | What is the level of education for the head of Household? | NO FORMAL EDUCATION……………11  RELIGIOUS (KHALWA or equivalent)………………………………12  PRIMARY/INTERMEDIATE/BASIC LEVEL  PARTIAL ………..……………21  COMPLETE ………………….22  SECONDARY  PARTIAL ……………………..31  COMPLETE ………………….32  ABOVE …………………………………...41 |  | |
| 10 | What is the main source of drinking water for members of your household?^1^ | PIPED WATER  PIPED INTO DWELLING 11  PIPED INTO YARD/PLOT 12  PUBLIC TAP/STANDPIPE 13  TUBE WELL OR BOREHOLE …...…… 21  DUG WELL  PROTECTED WELL 31  UNPROTECTED WELL 32  WATER FROM SPRING  PROTECTED SPRING 41  UNPROTECTED SPRING 42  RAINWATER …...……………………….. 51  TANKER TRUCK 61  CART WITH SMALL TANK 71  RIVERS/STREAMS/LAKES/DAMS……..81  POND………………………………………91  CANAL/IRRIGATION CHANNEL………101  BOTTLED WATER 96  OTHER 98  (SPECIFY) |  | |
| 10 A | DOES THE HOUSE HAS A CISTERN (even if it is made from local materials) FOR WATER STORAGE? | YES 11  NO 12 |  | |
| 11 | What kind of toilet facilities does your household use?^1^ | FLUSH OR POUR FLUSH TOILET  FLUSH TO PIPED SEWER  SYSTEM 11  FLUSH TO SEPTIC TANK …………. 12  FLUSH TO PIT LATRINE……………. 13  FLUSH TO SOMEWHERE ELSE . 14  FLUSH, DON’T KNOW WHERE . …. 15  PIT LATRINE  VENTILATED IMPROVED  PIT LATRINE (VIP).……………….. 21  PIT LATRINE WITH SLAB………… 22  PIT LATRINE WITHOUT SLAB/  OPEN PIT……………………………23  COMPOSTING TOILET………….………31  BUCKET TOILET…………………………41  HANGING TOILET/HANGING  LATRINE……………………………… 51  NO FACILITY/BUSH/FIELD……………..61  OTHER 96  (SPECIFY) |  | |
| 12 | Does your household have:^2^  Electricity?  A radio?  A television?  A mobile phone?  A refrigerator? | YES NO  ELECTRICITY 1 2  RADIO 1 2  TELEVISION 1 2  MOBILE PHONE 1 2  AIR CONDITION or A/C 1 2  REFRIGERATOR 1 2 |  | |
| 12A | HOW MANY MEMBERS OF THE HOUSE HOLD HAS SMART MOBILE PHONE (ONLY HOUSEHOLD MEMBERS, DON’T INCLUDE VISITORS OR OTHER NON FAMILY MEMBERS) | WRITE THE NUMBER  ______________ |  | |
| 13 | What type of fuel does your household mainly use for cooking? | ELECTRICITY 01  LPG/NATURAL GAS 02  KEROSENE 03  CHARCOAL 04  FIREWOOD/STRAW 05  DUNG 06  OTHER 96  (SPECIFY) |  | |
| 14 | MAIN MATERIAL OF THE FLOOR.  RECORD OBSERVATION. | NATURAL FLOOR  EARTH/SAND 11  DUNG 12  RUDIMENTARY FLOOR  WOOD PLANKS 21  PALM/BAMBOO 22  FINISHED FLOOR  PARQUET OR POLISHED WOOD 31  VINYL OR ASPHALT STRIPS 32  CERAMIC TILES 33  CEMENT 34  CARPET 35  OTHER 96  (SPECIFY) |  | |
| 15 | Does any member of your household own: | YES NO  BICYCLE 1 2  MOTORCYCLE/SCOOTER 1 2  CAR/TRUCK 1 2  CART………………………………1 2  DONKEY/CAMEL/HORSE……...1 2  OTHER…………………………….1 2 |  | |
| 16 | Does your household have any mosquito nets that can be used while sleeping? | YES............................................................1  NO..............................................................2 |  |  |
| 17 | How many mosquito nets does your household have? | NUMBER OF NETS [___] [___] |  |  |
| 18 | If there are NO nets in the household ask:  Why does the household not have nets  CIRCLE ONLY THE ONE BEST OTPTION | 1. Never heard of bed nets 2. Price of bed net is not affordable 3. nets NOT AVAILABLE in the area 4. Mosquito is not a problem in the area 5. Malaria is not a problem in the area 6. Nets do not protect against mosquitoes or malaria 7. Not enough space in the house to hang a net 8. The insecticide is not good for your health 9. we don’t like sleeping under a net |  |  |

| 19 | ASK RESPONDENT TO SHOW YOU THE NET(S) IN THE HOUSEHOLD.  IF MORE THAN THREE NETS, USE ADDITIONAL QUESTIONNAIRE(S). | NET # 1 | NET #2 | NET #3 |
| --- | --- | --- | --- | --- |
|  |  | OBSERVED ...................1  NOT OBSERVED......... 2 | OBSERVED ...................1  NOT OBSERVED......... 2 | OBSERVED ...................1  NOT OBSERVED......... 2 |
| 19 A | Was the net sealed in its plastic bag and not opened for use? | 1. YES 2. NO | 1. YES 2. NO | 1. YES 2. NO |
| 20 | How long ago did your household obtain the mosquito net? | ┌────┬────┐  MONTHS │░ ░░│░ ░│  AGO └────┴────┘  MORE THAN 3 YEARS AGO 95 | ┌────┬────┐  MONTHS │░ ░░│░ ░│  AGO └────┴────┘  MORE THAN 3 YEARS AGO 95 | ┌────┬────┐  MONTHS │░ ░░│░ ░│  AGO └────┴────┘  MORE THAN 3 YEARS AGO 95 |
| 21 | OBSERVE OR ASK THE BRAND OF MOSQUITO NET.  IF BRAND IS UNKNOWN, AND YOU CANNOT OBSERVE THE NET, SHOW PICTURES OF TYPICAL NET TYPES/BRANDS TO RESPONDENT. | LONG LASTING NET (e.g. OLYSET, PERMANET) …………11  NOT TREATED NET …………………………22  OTHER TYPE OF NET (LOCALY / HOME MADE)...….…..……….31  DON’T KNOW BRAND …98 | LONG LASTING NET (e.g. OLYSET, PERMANET) …………11  NOT TREATED NET …………………………22  OTHER TYPE OF NET (LOCALY / HOME MADE)...….…..……….31  DON’T KNOW BRAND …98 | LONG LASTING NET (e.g. OLYSET, PERMANET) …………11  NOT TREATED NET …………………………22  OTHER TYPE OF NET (LOCALY / HOME MADE)...….…..……….31  DON’T KNOW BRAND …98 |
| 22 | Did anyone sleep under this mosquito net last night? | YES ...............................1    NO .................................2  (SKIP TO 24) ───┤ NOT SURE ....................8  (SKIP TO 24) ───┤ | YES ...............................1    NO .................................2  (SKIP TO 24) ───┤ NOT SURE ....................8  (SKIP TO 24) ───┤ | YES ...............................1    NO .................................2  (SKIP TO 24) ───┤ NOT SURE ....................8  (SKIP TO 24) ───┤ |
| 23 | Who slept under this mosquito net last night?  RECORD THE RESPECTIVE LINE NUMBER FROM THE HOUSEHOLD SCHEDULE. | NAME ___________  ┌────┬────┐  LINE │░░ ░│░ │  NO └────┴────┘  NAME ___________  ┌────┬────┐  LINE │░░ ░│░ │  NO └────┴────┘  NAME ____________  ┌────┬────┐  LINE │░░ ░│░ │  NO └────┴────┘  NAME ____________  ┌────┬────┐  LINE │░░ ░│░ │  NO └────┴────┘  NAME ____________  ┌────┬────┐  LINE │░░ ░│░ │  NO └────┴────┘ | NAME ___________  ┌────┬────┐  LINE │░░ ░│░ │  NO └────┴────┘  NAME ___________  ┌────┬────┐  LINE │░░ ░│░ │  NO └────┴────┘  NAME ____________  ┌────┬────┐  LINE │░░ ░│░ │  NO └────┴────┘  NAME ____________  ┌────┬────┐  LINE │░░ ░│░ │  NO └────┴────┘  NAME ____________  ┌────┬────┐  LINE │░░ ░│░ │  NO └────┴────┘ | NAME ___________  ┌────┬────┐  LINE │░░ ░│░ │  NO └────┴────┘  NAME ___________  ┌────┬────┐  LINE │░░ ░│░ │  NO └────┴────┘  NAME ____________  ┌────┬────┐  LINE │░░ ░│░ │  NO └────┴────┘  NAME ____________  ┌────┬────┐  LINE │░░ ░│░ │  NO └────┴────┘  NAME ____________  ┌────┬────┐  LINE │░░ ░│░ │  NO └────┴────┘ |
| 24 |  | GO BACK TO 19 FOR NEXT NET; OR, IF NO MORE NETS, GO TO 25. | GO BACK TO 19 FOR NEXT NET; OR, IF NO MORE NETS, GO TO 25. | GO BACK TO 19 IN THE FIRST COLUMN OF NEW QUESTIONNAIRE IF THERE ARE MORE NETS TO BE OBSERVED; OR, IF NO MORE NETS, GO TO 25. |
| 25 | Has anyone sprayed the interior walls of your dwelling (walls NOT space) against mosquitoes at any time in the last 12 months? | 1. YES 2. NO 3. DON’T KNOW | | |
| 26 | Have you or any members of the household participated in discussion involving malaria protective measures or attended a public meeting or official meeting where Malaria was discussed in the last 6 months? | 1. YES 2. NO 3. DON’T KNOW | | |
| 27 | Have you or any members of the household come across printed materials or sign boards related to Malaria prevention and control in the last 6 months? | 1. YES 2. NO 3. DON’T KNOW | | |
| 28 | Have you received any malaria related information from the following?  Circle the appropriate options. | TV………………………………………………….1  Radio …………………………….………………..2  Newspaper ……………………….………………3  Other media (specify) ___________________4  No malaria related information received …......99 | | |
| 29 | Do you frequently listen to radio? | 1. YES 2. NO | | |
| 30 | Which channel do you frequently listen to? | 1. National Radio Station (*Um Durman*) 2. FM 100 (*Sudanese House*) 3. Police Voice (*Saheroon*) 4. Military Voice 5. KRT FM (Economic) 6. Station Four 7. FM 96 (*hala*) 8. Medical Station 9. Darfur station 10. State Station 11. Sport Station 12. Other (specify)____________________________________________ | | |
| 31 | What hours do you listen to this channel? | 1. Very early morning [5.00 – 7.00 am] 2. Early morning [7.00 – 8.00 am] 3. Mid- morning [8.00 – 10.00 am] 4. Pre-afternoon [10.00 am – 12.00 pm] 5. Afternoon [12.00 – 15.00 pm] 6. Very early evening [15.00 – 17.00 pm] 7. Early evening [17.00 – 19.00 pm] 8. Mid evening [19.00 – 20.00 pm] 9. Late evening [after 20.00 pm] | | |
| 32 | Have you or anyone of your family seen a man with the worm in this photo during the last 6 months?  SHOW THE HOUSEHOLD HEAD AND MEMBERS THE GUENEA WORM PHOTO | 1. YES 2. NO | | |
| 33. | What is the type of health facility that the family use for consultation, health care and disease management? | 1. Public Health Facility 2. Private Health Facility / private clinic 3. NGO Health Facility 4. Other | | |
| 34 | Have you or anyone of your family been admitted to a hospital for more than 24 hours during the last 6 months? | 1. YES 2. NO   If NO skip to 39 | | |
| 35 | If yes to 34 above, where have you or your family member been admitted? for the LAST ADMISSION | 1. A hospital within the state of my residence 2. A hospital in another state 3. A hospital outside the country | | |
| 36 | If have been admitted to a hospital in another state or outside the country in 35 above, why did you chose this? for the LAST ADMISSION | 1. Service I need not available 2. Service not satisfying my expectation 3. Waiting time is too much 4. To make sure of the diagnosis 5. Cost of alternate service was affordable (in the place where I went) 6. Other reason, specify _____________________ | | |
| 37 | What is the outcome of that admission? for the LAST ADMISSION | 1. Survived and Cured 2. Survived and Cured with residual 3. Survived but Not cured 4. Died / death | | |
| 38 | Has any member of your family died within the last 2 years? | 1. YES 2. NO | | |
| 38 A | If YES to 38 above, was the death during this year or the year before? | 1. This year 2. The previous year | | |
| 39 | If YES to 38 above, was death associated with fever? | 1. YES 2. NO | | |
| 40 | If YES to 38 above, was death associated with malaria? | 1. YES 2. NO | | |
| 41 | If YES to 38 above, what was his/her gender? | 1. Male 2. Female | | |
| 42 | If YES to 38 above, what was his/her age? | WRITE THE AGE OF THE DECEASED IN YEARS, IF LESS THAN 1 YEAR WRITE 00  [___] [___] Years | | |
| 43 | If she was a female, between the age of 15 – 49 years, was she dead during pregnancy, labor or puerperium? | 1. No 2. Yes, During Pregnancy 3. Yes, During Labor 4. Yes, During Puerperium | | |
| 44 | If YES to 38 above, where was the death occur? | 1. At Home 2. On the Way to the Hospital 3. At the Health Facility | | |
